# Demographic, socioeconomic, and health correlates of unmet need for mental health treatment in the United States, 2002–16: evidence from the national surveys on drug use and health

**DOI:** 10.1186/s12939-019-1026-y

**Published:** 2019-08-05

**Authors:** Justin C. Yang, Andres Roman-Urrestarazu, Martin McKee, Carol Brayne

**Affiliations:** 1 Department of Public Health & Primary Care, Institute of Public Health, University of Cambridge, Forvie Site, Robinson Way, Cambridge, CB2 0SR UK; 2 Department of Health Services Research and Policy, London School of Hygiene & Tropical Medicine, London, UK

**Keywords:** Mental health treatment, Unmet need

## Abstract

**Background:**

Unmet need for mental health services remains high in the United States and is disproportionately concentrated in some groups. The scale and nature of these disparities have not been fully elucidated and bear further scrutiny. As such, in this study, we examine the demographic, socioeconomic, and health correlates of unmet need for mental health treatment as well as the reasons for unmet need.

**Methods:**

We draw upon the National Survey for Drug Use and Health (NSDUH) from 2002 to 16 for adults aged 18 and over in the United States (*n* = 579,017). Using multivariable logistic regression, we simultaneously model the demographic, socioeconomic, and health correlates of unmet need for mental health treatment from 2002 to 16. We also analyse the reasons for unmet need expressed by these populations, reasons which include cost, perceived stigma, minimisation of symptoms, low perceived effectiveness of treatment, and structural barriers.

**Results:**

Major characteristics associated with increased odds of unmet need include past year substance abuse or dependence (other than hallucinogens and sedatives), fair, poor, or very poor health, being female, and an educational attainment of college or higher. With respect to reasons for unmet need, cost was most often cited, followed by perceived stigma, structural barriers, and minimisation. Characteristics associated with increased odds of indicating cost as a reason for unmet need include: being uninsured or aged 26–35. Minimisation and low perceived effectiveness are mentioned by high-income persons as reasons for unmet need. College-educated persons and women had higher odds of citing structural barriers as a reason for unmet need.

**Conclusions:**

The correlates and causes of unmet need highlight the intersectionality of individual health needs with implications on addressing inequities in mental health policy and practice.

## Introduction

Mental disorders and substance use disorders are major contributors to years lived with disability in the United States (3,536,895.4 years lived with disability [YLDs] in 2016, or 1095.45 YLDs per 100,000 population) [1]. They are common, with data from the National Survey on Drug Use and Health (2010–2012) finding that 18.4% of adults had a mental illness and 8.6% reported substance abuse/dependence, while 2.2% had both [2]. Federal policymakers have responded with a variety of legislative approaches, notably the *Mental Health Parity and Addiction Equity Act of 2008* (MHPAEA), while the *Patient Protection and Affordable Care Act of 2010* (ACA) includes specific provisions for mental health [3]. Yet, despite these policies, there are large and persisting disparities in access to and receipt of mental health services. Individual studies have identified a range of underserved populations, which include certain ethnic minorities [4] and those lacking insurance [5]. Moreover, difficulties in accessing adequate mental health treatment have been documented among individuals with substance use disorders [6]. These previous studies signal an urgent need to address disparities in mental health treatment.

However, describing those whose needs for care are unmet is only a first step. It is also necessary to understand the reasons, if an appropriate policy response is to be developed. Possible explanations may lie within the affected individual or within the health care system.

In this study, we seek to characterise the demographic, socioeconomic, and health correlates of unmet need for mental health care among Americans, using data from the period 2002 to 2016 and to understand the reasons their needs are not being met. To our knowledge, this is the first attempt to simultaneously analyse these correlates at once. With respect to the reasons for unmet need, we draw upon and extend existing analyses which have reviewed these causes which include: cost, perceived stigma, minimisation of symptoms, low perceived effectiveness of treatment, and structural barriers [5].

## Methods

This study utilises data from the 2002–16 waves of the NSDUH, a nationally representative, annual household survey of civilian, non-institutionalised individuals aged 12 and above in the United States [7–21].The NSDUH collects data on measures of mental health, including substance use and unmet need for mental health treatment, as well as a range of demographic, socioeconomic, and health-related variables [7–21]. Households are selected from all 50 states and the District Columbia, excluding individuals with no fixed address, active duty military personnel, and individuals living in institutionalised facilities [7–21]. Sampling and analytical weights for the NSDUH datasets were provided by the Substance Abuse and Mental Health Services Administration (SAMHSA) to address unit- and individual-level non-response [7–21]. In the current study, the only exclusion criterion we applied to the dataset was to exclude respondents aged 17 or below given our interest in the adult population exclusively.

Age at time of survey was categorised as: 18–25, 26–34, 35–49, and 50 or over. Sex was coded as either male or female. Ethnicity was coded as non-Hispanic White, non-Hispanic Black, non-Hispanic Native American or Alaskan Native, non-Hispanic Native Hawaiian or Pacific Islander, non-Hispanic Asian, non-Hispanic mixed, or Hispanic. Marital status was coded as never married, married, widowed, divorced, or separated. Population density was coded as metropolitan statistical area (MSA) or core based statistical area (CBSA) 1 million or more (“urban”), MSA or CBSA with fewer than 1 million (“suburban”), or non-MSA/CBSA (“rural”) based on data from the 2000 or 2010 census and information from the Core Based Statistical Area classifications compiled by the Office of Management and Budget (OMB) [7–21].

Education was coded as the highest level reached from among elementary school, middle school, high school or college and higher. Employment was coded as full-time employed, part-time employed, unemployed, or other (defined as those not in the labour force such as students, retirees, or disabled individuals). Annual household income was coded as less than $20,000, between $20,000 and $49,999, between $50,000 and $74,999, and $75,000 or greater. A dichotomous variable was created to indicate whether the respondent was a recipient of a government assistance program (i.e. Supplemental Security Income [SSI], food stamps, cash assistance, and/or non-cash assistance). Insurance provider was coded as privately insured, insured by Medicare, insured by Medicaid, insured by Tricare or Veterans Administration (VA), uninsured, or other.

Self-rated health was dichotomised into two categories: those reporting excellent, very good, or good self-rated health and those reporting fair, poor, or very poor self-rated health. Dichotomous variables indicating past-year substance abuse or dependence were coded for each of: alcohol, pain relievers, cocaine, hallucinogens, heroin, inhalants, marijuana, sedatives, stimulants, and tranquilizers.

Expressed unmet need for mental health treatment (“unmet need”) was defined as meeting both the following conditions: (a) perceiving of a need for mental health treatment in the past year and (b) perceiving receipt of insufficient mental health treatment in the past year, including those adults who had received mental health treatment in the past year but perceived a further need that was unmet [7–21]. A dichotomous variable was created to code for responses to the question, “During the past 12 months, was there any time when you needed mental health treatment or counselling for yourself but didn’t get it?” [7–21]. Among individuals expressing unmet need for mental health treatment, further dichotomous variables were coded to indicate whether the unmet need was due to one or more of the following reasons: cost, stigma, minimisation, low perceived treatment effectiveness, structural barriers or other reasons using definitions set by SAMHSA [7–21] and from established literature [5]. The specification of these variables are shown in Table 1.

**Table 1:** Reasons for unmet need and corresponding survey responses

Reasons for Unmet Need	Survey Responses
Cost	Could not afford the costs of treatment
	Health insurance does not cover mental health treatment
	Health insurance does not cover enough for mental health treatment
Stigma	Might cause neighbours to have a negative opinion
	Might have a negative effect on job
	Did not want others to find out
	Concerned about confidentiality
	Concerned about being committed/medicated
Minimisation	Did not feel that they needed mental health treatment
	Felt that they could handle the problem without treatment
Low Perceived Effectiveness of Treatment	Did not feel that mental health treatment would help
Structural Barriers	Did not know where to go for mental health treatment
	Did not have the time
	Inconvenient to attend mental health treatment
Other Reasons	Other reason than those listed above

Item non-response was addressed using imputation based on predictive mean neighbourhood (PMN), as has been done with NSDUH datasets since 1999 [7–21]. Imputation is performed most for variables pertaining to ethnicity and government assistance, drawing upon responses to other related questions where logical errors and item non-response exist, and to a lesser degree for education, marital status, income, and health insurer [7–21]. The percentage of observations with imputed values ranges from < 0.1 to 4% based on variable [7–21].

Statistical analyses were performed in Stata 14. We conducted bivariate descriptive analysis of our primary dichotomous variable of interest, unmet need for mental health treatment, and multiple maximum-likelihood logit regression with weighted least squares on social, economic, and health correlates, with and without adjustment, to assess the demographic, socioeconomic, and health correlates of unmet mental health treatment both individually and simultaneously adjusting for all other correlates. Further analyses of our secondary dichotomous variables of interest indicating reason(s) for unmet need were also conducted using multiple maximum-likelihood logit regressions with weighted least squares on social, economic, and health correlates. Given the NSDUH’s complex sampling design, all analyses were weighted using analytical weights provided by SAMHSA with each annual dataset.

## Results

Descriptive characteristics of our sample are shown in Table 2. From 2002 to 16, between 36,000–43,000 adults aged 18 or above were surveyed annually, yielding a total study population of 579,017. Table 3 shows the reasons respondents gave for past-year unmet need for mental health treatment from 2002 to 16.

**Table 2:** Sample characteristics for adults age 18 and over by percentage (%), 2002–16

Year	2002	2003	2004	2005	2006	2007	2008	2009	2010	2011	2012	2013	2014	2015	2016
*n*	*36370*	*37026*	*37308*	*37227*	*36965*	*37708*	*37504*	*37707*	*38919*	*39133*	*37869*	*37424*	*41671*	*43561*	*42625*
Age															
18–25	48.7%	49.6%	49.5%	49.6%	48.5%	48.6%	50.4%	49.8%	49.0%	49.0%	49.2%	48.5%	31.4%	33.4%	32.0%
26–34	15.2%	15.0%	15.3%	14.8%	14.9%	15.5%	14.8%	14.9%	15.1%	14.4%	14.5%	14.6%	20.1%	20.9%	20.5%
35–49	22.8%	21.9%	21.6%	21.8%	20.8%	21.8%	20.8%	20.7%	21.4%	19.5%	19.5%	20.1%	27.0%	25.6%	26.7%
> 50	13.2%	13.5%	13.6%	13.8%	15.8%	14.2%	14.1%	14.7%	14.5%	17.1%	16.8%	16.9%	21.5%	20.1%	20.8%
Female	53.5%	53.1%	53.4%	54.0%	53.0%	53.5%	53.5%	53.3%	53.3%	52.8%	53.1%	54.0%	53.4%	54.5%	53.4%
Ethnicity															
Non-Hispanic White	70.4%	67.7%	66.6%	65.6%	65.8%	65.6%	63.3%	63.2%	64.0%	62.9%	62.0%	61.1%	62.4%	59.7%	60.9%
Non-Hispanic Black	11.6%	11.7%	11.9%	11.9%	12.1%	11.6%	12.4%	12.3%	12.1%	12.8%	12.7%	13.0%	11.8%	12.6%	12.8%
Non-Hispanic Native American or Alaskan Native	1.1%	1.3%	1.3%	1.5%	1.4%	1.4%	1.5%	1.4%	1.6%	1.6%	1.4%	1.4%	1.6%	1.5%	1.5%
Non-Hispanic Native Hawaiian or Pacific Islander	0.5%	0.4%	0.4%	0.5%	0.5%	0.5%	0.5%	0.4%	0.5%	0.4%	0.6%	0.6%	0.5%	0.5%	0.5%
Non-Hispanic Asian	2.9%	3.2%	3.5%	3.5%	3.2%	3.7%	3.8%	3.9%	3.8%	3.6%	4.1%	4.4%	4.4%	4.7%	4.4%
Non-Hispanic Mixed	1.6%	2.0%	2.3%	2.3%	2.3%	2.5%	2.5%	2.9%	2.7%	3.0%	2.9%	3.1%	3.0%	3.3%	3.2%
Hispanic	11.9%	13.8%	14.0%	14.7%	14.7%	14.7%	15.9%	15.8%	15.3%	15.6%	16.2%	16.5%	16.2%	17.6%	16.6%
Marital Status															
Never married	48.3%	49.1%	49.6%	50.3%	49.7%	50.5%	52.8%	53.1%	53.0%	52.9%	53.3%	53.7%	42.7%	43.7%	44.8%
Married	40.1%	39.4%	38.7%	38.0%	38.8%	37.9%	35.9%	35.6%	35.4%	35.1%	34.6%	34.4%	42.7%	41.4%	41.0%
Widowed	2.5%	2.3%	2.4%	2.4%	2.8%	2.4%	2.3%	2.3%	2.5%	2.5%	2.6%	2.5%	3.1%	3.7%	3.0%
Divorced or Separated	9.1%	9.2%	9.3%	9.3%	8.7%	9.2%	8.9%	9.0%	9.2%	9.4%	9.5%	9.3%	11.5%	11.1%	11.2%
MSA/CBSA Size															
1 million or more	34.8%	34.1%	34.4%	42.1%	42.1%	41.7%	42.1%	42.5%	41.9%	40.7%	41.5%	42.4%	43.1%	43.2%	42.5%
Fewer than 1 million	38.9%	39.0%	38.3%	49.2%	48.9%	49.9%	49.4%	49.7%	50.4%	51.1%	50.6%	49.6%	49.0%	49.3%	49.9%
Not a MSA/CBSA	26.3%	26.9%	27.2%	8.7%	9.0%	8.4%	8.5%	7.8%	7.7%	8.2%	7.9%	7.9%	7.8%	7.5%	7.6%
Educational Attainment															
Elementary School	0.9%	1.1%	1.0%	1.1%	1.1%	1.2%	1.1%	1.0%	0.9%	0.9%	1.0%	0.9%	1.1%	0.9%	0.9%
Middle School	2.8%	3.0%	2.9%	3.2%	3.0%	2.9%	2.6%	2.6%	2.6%	2.4%	2.2%	2.3%	2.4%	2.5%	2.0%
High School	47.5%	48.8%	48.1%	47.3%	47.3%	46.2%	47.4%	45.9%	45.2%	45.0%	45.2%	43.7%	40.2%	38.1%	36.6%
College or Higher	48.8%	47.0%	48.0%	48.5%	48.5%	49.7%	48.9%	50.5%	51.2%	51.7%	51.6%	53.0%	56.4%	58.5%	60.6%
Employment Status															
Full Time	56.0%	53.4%	53.1%	54.3%	54.3%	54.1%	52.1%	46.8%	47.1%	46.4%	47.5%	47.8%	52.9%	50.9%	51.7%
Part Time	17.9%	18.6%	18.5%	18.3%	17.9%	18.2%	18.7%	19.8%	19.7%	19.4%	19.1%	19.7%	16.4%	16.1%	15.8%
Unemployed	5.1%	6.2%	5.8%	5.4%	5.5%	5.6%	6.9%	10.1%	9.6%	9.2%	8.9%	8.1%	6.1%	6.6%	6.1%
Other (including not in labour force)	21.0%	21.8%	22.6%	22.0%	22.3%	22.2%	22.4%	23.3%	23.6%	25.0%	24.4%	24.3%	24.6%	26.4%	26.3%
Annual Household Income															
Less than $20,000	25.0%	26.4%	27.5%	26.2%	25.7%	24.8%	24.4%	25.1%	26.2%	26.6%	26.1%	26.1%	22.2%	22.3%	21.0%
$20,000–$49,999	39.5%	38.8%	37.6%	36.9%	36.1%	35.1%	34.9%	34.6%	33.9%	34.6%	34.6%	33.2%	31.8%	32.2%	31.7%
$50,000–$74,999	16.8%	16.4%	15.9%	16.3%	16.1%	16.9%	17.2%	16.1%	15.9%	15.0%	15.2%	15.5%	16.4%	15.5%	15.4%
$75,000 or more	18.7%	18.4%	19.1%	20.6%	22.1%	23.2%	23.5%	24.2%	24.1%	23.8%	24.2%	25.2%	29.6%	30.0%	32.0%
Receives Government Assistance	13.7%	15.9%	17.2%	18.0%	17.5%	17.7%	18.1%	19.8%	21.7%	24.5%	25.7%	25.9%	22.9%	23.0%	21.6%
Self-Rated Health															
Good, very good, or excellent	92.1%	91.6%	91.0%	90.7%	90.6%	90.5%	90.5%	90.5%	90.6%	90.0%	89.8%	90.0%	88.6%	88.5%	88.8%
Fair, poor, or very poor	7.9%	8.4%	9.0%	9.3%	9.4%	9.5%	9.5%	9.5%	9.4%	10.0%	10.2%	10.0%	11.4%	11.5%	11.2%
Health Insurer															
Private	69.2%	65.5%	64.3%	63.3%	63.4%	62.2%	61.2%	59.7%	59.5%	59.1%	59.6%	60.2%	63.6%	62.6%	63.9%
Medicare	2.9%	2.7%	2.8%	3.0%	3.1%	3.0%	3.3%	3.3%	3.3%	4.0%	4.0%	3.9%	5.1%	4.9%	4.9%
Medicaid	6.9%	8.0%	8.6%	9.0%	8.5%	9.0%	9.8%	10.2%	10.7%	11.4%	11.3%	11.6%	11.7%	14.3%	14.7%
Tricare or VA	1.4%	1.7%	1.6%	1.7%	1.6%	1.6%	1.7%	1.8%	2.0%	2.1%	2.1%	2.1%	2.1%	2.0%	2.2%
Other	1.8%	2.4%	2.4%	2.4%	2.5%	2.8%	2.6%	2.8%	2.9%	2.8%	3.0%	2.9%	3.0%	3.7%	3.0%
Uninsured	17.7%	19.8%	20.2%	20.7%	21.0%	21.5%	21.4%	22.2%	21.7%	20.6%	20.0%	19.3%	14.6%	12.5%	11.4%
Past-Year Substance Abuse or Dependence															
Alcohol	12.8%	12.4%	12.6%	12.6%	12.3%	12.1%	12.2%	12.0%	11.4%	10.7%	10.5%	9.9%	8.4%	7.9%	7.7%
Pain Reliever	0.9%	0.8%	0.9%	1.1%	1.0%	1.2%	1.3%	1.3%	1.2%	1.2%	1.3%	1.1%	0.9%	1.0%	0.9%
Cocaine	1.0%	1.0%	1.1%	1.1%	1.0%	1.0%	0.9%	0.7%	0.5%	0.5%	0.5%	0.5%	0.4%	0.4%	0.4%
Hallucinogen	0.4%	0.3%	0.3%	0.3%	0.3%	0.3%	0.3%	0.3%	0.3%	0.2%	0.2%	0.2%	0.1%	0.2%	0.2%
Heroin	0.1%	0.1%	0.2%	0.2%	0.2%	0.2%	0.2%	0.3%	0.2%	0.3%	0.4%	0.4%	0.3%	0.3%	0.4%
Inhalant	0.1%	0.1%	0.1%	0.1%	0.1%	0.1%	0.1%	0.1%	0.1%	0.1%	< 0.1%	< 0.1%	< 0.1%	< 0.1%	< 0.1%
Marijuana	3.4%	3.5%	3.6%	3.3%	3.3%	3.2%	3.4%	3.3%	3.2%	3.1%	3.2%	3.0%	2.3%	2.3%	2.3%
Sedative	0.1%	0.1%	0.1%	0.1%	0.1%	0.1%	0.1%	0.1%	0.1%	< 0.1%	0.1%	0.1%	0.1%	0.1%	0.1%
Stimulant	0.3%	0.3%	0.3%	0.3%	0.2%	0.3%	0.2%	0.3%	0.2%	0.2%	0.3%	0.3%	0.3%	0.3%	0.3%
Tranquilizer	0.3%	0.3%	0.4%	0.3%	0.3%	0.3%	0.3%	0.3%	0.3%	0.2%	0.3%	0.3%	0.2%	0.3%	0.3%
Past-Year Unmet Need for Mental Health Treatment	7.4%	7.1%	7.2%	7.2%	6.4%	6.8%	6.8%	7.1%	6.6%	6.6%	6.7%	6.6%	6.4%	6.3%	6.8%

**Table 3:** Reason(s) for unmet need among adults reporting unmet need for mental health treatment in the past year, 2002–16

Year	2002	2003	2004	2005	2006	2007	2008	2009	2010	2011	2012	2013	2014	2015	2016
*n*	*2680*	*2640*	*2665*	*2678*	*2368*	*2557*	*2530*	*2687*	*2556*	*2564*	*2537*	*2463*	*2652*	*2718*	*2891*
Reason for Unmet Need															
Cost	61.8%	62.3%	63.8%	63.0%	47.3%	47.3%	48.7%	50.8%	50.9%	51.6%	48.4%	48.5%	48.5%	44.5%	43.8%
Perceived Stigma	31.2%	32.1%	30.8%	30.2%	29.7%	31.8%	31.1%	30.5%	32.1%	29.5%	31.0%	30.2%	30.4%	32.8%	34.9%
Minimisation	13.8%	34.8%	34.5%	32.3%	31.5%	32.5%	30.3%	30.6%	30.0%	27.9%	29.9%	29.2%	28.4%	31.6%	33.0%
Low Perceived Effectiveness	1.6%	11.8%	11.3%	10.8%	10.4%	11.8%	10.0%	11.0%	10.9%	10.3%	10.3%	9.9%	10.1%	12.6%	11.6%
Structural Barriers	28.8%	34.8%	32.9%	31.7%	29.9%	31.9%	32.5%	31.9%	32.1%	32.2%	33.6%	35.8%	35.8%	41.8%	42.2%
Other Reason(s)	4.4%	8.3%	7.4%	7.9%	7.3%	7.8%	7.4%	7.6%	7.3%	7.7%	7.2%	7.9%	8.3%	9.2%	9.7%

### Unmet need

Unadjusted and adjusted odds of reporting unmet need among those perceiving a need for mental health treatment in the past year according to a range of demographic, socioeconomic, and health characteristics are shown in Table 4. Factors increasing the odds of reported unmet need included: being female, attaining an educational level of college or higher, receiving government assistance, reporting fair, poor, or very poor health, and being insured by Medicaid, Tricare or VA, or not having health insurance. In addition, with the exception of hallucinogens and sedatives, past-year abuse or dependence on any substance increased the odds of unmet need. On the other hand, attributes of decreased odds of unmet need included: age over 34, being married or widowed, and having a household income of $50,000 or more.

**Table 4:** Odds ratios and associated 95% confidence intervals for unadjusted and adjusted multiple logistic regression of demographic, socioeconomic, and health characteristics associated with unmet need for mental health treatment for adults, 2002–16

	Unadjusted		Adjusted	
Age				
18–25 years old	1		1	
26–34 years old	0.886	(0.853–0.920)	1.056	(1.007–1.108)
35–49 years old	0.698	(0.675–0.722)	0.895	(0.851–0.941)
50 or older	0.293	(0.277–0.311)	0.364	(0.336–0.395)
Female	2.031	(1.952–2.113)	2.299	(2.204–2.399)
Ethnicity				
Non-Hispanic White	1		1	
Non-Hispanic Black	0.840	(0.789–0.895)	0.568	(0.530–0.610)
Non-Hispanic Native American or Alaskan Native	1.315	(1.135–1.525)	0.795	(0.676–0.934)
Non-Hispanic Native Hawaiian or Pacific Islander	0.640	(0.464–0.881)	0.478	(0.339–0.672)
Non-Hispanic Asian	0.405	(0.351–0.467)	0.376	(0.326–0.433)
Non-Hispanic Mixed	1.697	(1.497–1.925)	1.244	(1.094–1.416)
Hispanic	0.734	(0.693–0.777)	0.547	(0.510–0.586)
Marital Status				
Never married	1		1	
Married	0.428	(0.413–0.445)	0.755	(0.716–0.797)
Widowed	0.320	(0.282–0.364)	0.612	(0.528–0.709)
Divorced or Separated	0.960	(0.914–1.009)	1.228	(1.154–1.306)
MSA/CBSA Size				
1 million or more	1		1	
Fewer than 1 million	1.090	(1.056–1.125)	0.983	(0.951–1.016)
Not a MSA/CBSA	0.946	(0.895–1.001)	0.854	(0.801–0.911)
Educational Attainment				
Elementary School	1		1	
Middle School	1.307	(0.972–1.758)	1.093	(0.804–1.487)
High School	1.709	(1.322–2.211)	1.301	(0.991–1.708)
College or Higher	2.062	(1.600–2.658)	2.043	(1.563–2.671)
Employment Status				
Full Time	1		1	
Part Time	1.552	(1.478–1.629)	1.175	(1.115–1.239)
Unemployed	1.914	(1.784–2.052)	1.226	(1.136–1.323)
Other (including not in labour force)	1.118	(1.069–1.169)	1.128	(1.069–1.190)
Annual Household Income				
Less than $20,000	1		1	
$20,000–$49,999	0.687	(0.659–0.717)	0.953	(0.907–1.000)
$50,000–$74,999	0.580	(0.551–0.610)	0.897	(0.842–0.955)
$75,000 or more	0.475	(0.452–0.498)	0.785	(0.740–0.833)
Receives Government Assistance	2.045	(1.969–2.123)	1.461	(1.389–1.537)
Self-Rated Health				
Good, very good, or excellent	1		1	
Fair, poor, or very poor	1.985	(1.905–2.069)	2.433	(2.320–2.552)
Health Insurer				
Private	1		1	
Medicare	0.992	(0.910–1.082)	1.074	(0.965–1.197)
Medicaid	2.452	(2.336–2.574)	1.196	(1.118–1.280)
Tricare or VA	1.662	(1.480–1.867)	1.573	(1.388–1.783)
Other	1.630	(1.462–1.816)	1.086	(0.965–1.221)
Uninsured	1.831	(1.750–1.917)	1.310	(1.241–1.382)
Past-Year Substance Abuse or Dependence				
Alcohol	3.629	(3.471–3.795)	2.628	(2.499–2.763)
Pain Relievers	8.443	(7.697–9.261)	2.616	(2.304–2.970)
Cocaine	7.340	(6.651–8.101)	2.056	(1.785–2.369)
Hallucinogens	8.146	(6.778–9.790)	1.266	(0.957–1.675)
Heroin	7.656	(6.485–9.038)	1.471	(1.112–1.946)
Inhalants	9.192	(6.234–13.55)	2.325	(1.351–4.002)
Marijuana	4.678	(4.383–4.993)	1.955	(1.800–2.123)
Sedatives	16.73	(11.90–23.51)	1.546	(0.832–2.873)
Stimulants	12.77	(10.60–15.37)	2.156	(1.623–2.864)
Tranquilizers	12.35	(10.37–14.70)	1.834	(1.392–2.418)

### Reasons for unmet need

Table 5 shows the results of adjusted logistic regression of individual demographic, socioeconomic, and health characteristics associated reported reasons for unmet need among respondents indicating past-year unmet need for mental health treatment.

**Table 5:** Odds ratios and associated 95% confidence intervals for adjusted multiple logistic regression of demographic, socioeconomic, and health characteristics associated with reasons for past-year unmet need for mental health treatment for adults, 2002–16

Cost			Perceived Stigma		Minimisation		Low Perceived Effectiveness		Structural Barriers		Other	
Age												
18–25 years old	1		1		1		1		1		1	
26–34 years old	1.413	(1.294–1.543)	0.674	(0.615–0.739)	0.755	(0.685–0.832)	0.716	(0.618–0.829)	0.878	(0.804–0.959)	1.139	(0.970–1.337)
35–49 years old	1.486	(1.341–1.647)	0.575	(0.515–0.642)	0.649	(0.583–0.723)	0.612	(0.519–0.721)	0.724	(0.658–0.797)	1.245	(1.070–1.448)
50 or older	1.121	(0.981–1.282)	0.380	(0.320–0.452)	0.706	(0.608–0.819)	0.688	(0.535–0.884)	0.585	(0.498–0.688)	1.388	(1.074–1.795)
Female	1.069	(0.993–1.150)	0.748	(0.695–0.806)	1.015	(0.928–1.111)	0.893	(0.782–1.019)	1.268	(1.177–1.366)	0.965	(0.836–1.115)
Ethnicity												
Non-Hispanic White	1		1		1		1		1		1	
Non-Hispanic Black	0.700	(0.611–0.803)	1.070	(0.946–1.211)	1.086	(0.939–1.255)	0.673	(0.538–0.841)	0.890	(0.783–1.012)	0.803	(0.621–1.038)
Non-Hispanic Native American or Alaskan Native	0.536	(0.410–0.701)	1.070	(0.789–1.452)	0.904	(0.613–1.334)	0.597	(0.349–1.021)	1.007	(0.729–1.391)	1.361	(0.808–2.293)
Non-Hispanic Native Hawaiian or Pacific Islander	0.734	(0.369–1.460)	2.060	(0.926–4.581)	1.861	(0.910–3.804)	1.162	(0.542–2.492)	1.431	(0.652–3.140)	0.882	(0.354–2.201)
Non-Hispanic Asian	0.835	(0.620–1.123)	1.185	(0.948–1.482)	1.101	(0.869–1.394)	1.044	(0.687–1.588)	1.349	(1.059–1.716)	0.858	(0.504–1.461)
Non-Hispanic Mixed	0.889	(0.707–1.116)	0.951	(0.768–1.179)	0.918	(0.723–1.167)	1.026	(0.790–1.331)	1.148	(0.900–1.465)	1.853	(1.262–2.722)
Hispanic	0.740	(0.653–0.839)	1.059	(0.929–1.209)	0.993	(0.873–1.131)	0.691	(0.548–0.871)	1.015	(0.897–1.148)	0.893	(0.717–1.111)
Marital Status												
Never married	1		1		1		1		1		1	
Married	0.948	(0.859–1.046)	0.944	(0.847–1.052)	1.050	(0.947–1.165)	0.749	(0.645–0.869)	1.034	(0.943–1.134)	0.776	(0.635–0.949)
Widowed	0.847	(0.649–1.107)	0.743	(0.536–1.030)	0.930	(0.665–1.300)	0.579	(0.310–1.083)	0.812	(0.582–1.131)	0.685	(0.446–1.052)
Divorced or Separated	1.119	(0.998–1.255)	0.914	(0.797–1.047)	0.978	(0.854–1.120)	0.791	(0.639–0.978)	0.979	(0.858–1.116)	0.916	(0.750–1.119)
MSA/CBSA Size												
1 million or more	1		1		1		1		1		1	
Fewer than 1 million	0.989	(0.916–1.068)	1.169	(1.081–1.264)	1.084	(1.007–1.167)	1.044	(0.920–1.185)	0.845	(0.780–0.915)	1.136	(0.975–1.322)
Not a MSA/CBSA	0.934	(0.828–1.052)	1.445	(1.266–1.649)	1.167	(1.023–1.331)	0.985	(0.768–1.263)	0.915	(0.812–1.032)	0.836	(0.638–1.095)
Educational Attainment												
Elementary School	1		1		1		1		1		1	
Middle School	1.083	(0.605–1.939)	1.085	(0.570–2.066)	1.203	(0.465–3.108)	0.879	(0.247–3.121)	1.153	(0.654–2.034)	2.015	(0.925–4.389)
High School	1.095	(0.648–1.851)	0.941	(0.525–1.687)	1.437	(0.593–3.486)	0.893	(0.318–2.508)	1.629	(1.034–2.568)	4.072	(1.911–8.678)
College or Higher	1.178	(0.703–1.976)	1.002	(0.554–1.810)	1.672	(0.691–4.045)	1.109	(0.397–3.100)	2.166	(1.379–3.400)	5.125	(2.389–10.99)
Employment Status												
Full Time	1		1		1		1		1		1	
Part Time	1.052	(0.957–1.155)	0.885	(0.796–0.983)	1.012	(0.928–1.102)	1.012	(0.893–1.147)	0.880	(0.801–0.966)	1.369	(1.161–1.615)
Unemployed	1.127	(0.977–1.299)	0.932	(0.812–1.070)	0.795	(0.687–0.920)	0.794	(0.615–1.026)	0.838	(0.723–0.971)	1.531	(1.207–1.943)
Other (including not in labour force)	0.804	(0.732–0.883)	0.921	(0.833–1.019)	1.041	(0.935–1.159)	1.118	(0.967–1.292)	0.906	(0.820–1.000)	1.943	(1.649–2.290)
Annual Household Income												
Less than $20,000	1		1		1		1		1		1	
$20,000–$49,999	1.096	(0.995–1.207)	0.951	(0.869–1.040)	0.960	(0.861–1.070)	0.962	(0.830–1.115)	0.897	(0.821–0.980)	1.053	(0.890–1.245)
$50,000–$74,999	0.881	(0.786–0.988)	1.009	(0.891–1.142)	1.074	(0.951–1.212)	1.232	(0.997–1.522)	0.947	(0.846–1.060)	0.913	(0.714–1.167)
$75,000 or more	0.569	(0.511–0.634)	1.045	(0.923–1.183)	1.340	(1.190–1.509)	1.224	(1.014–1.478)	0.984	(0.883–1.098)	1.105	(0.875–1.395)
Receives Government Assistance	1.005	(0.906–1.116)	0.982	(0.886–1.090)	0.872	(0.783–0.972)	0.933	(0.800–1.088)	1.109	(1.001–1.227)	1.048	(0.893–1.230)
Self-Rated Health												
Good, very good, or excellent	1		1		1		1		1		1	
Fair, poor, or very poor	1.245	(1.126–1.375)	1.133	(1.023–1.256)	0.750	(0.668–0.842)	1.011	(0.831–1.230)	0.898	(0.815–0.989)	1.101	(0.925–1.310)
Health Insurer												
Private	1		1		1		1		1		1	
Medicare	0.860	(0.729–1.013)	1.141	(0.922–1.413)	0.649	(0.506–0.833)	0.703	(0.473–1.047)	0.958	(0.801–1.147)	1.119	(0.834–1.501)
Medicaid	0.795	(0.700–0.902)	1.095	(0.968–1.238)	0.801	(0.694–0.924)	0.996	(0.809–1.227)	1.038	(0.908–1.187)	1.207	(0.984–1.480)
Tricare or VA	0.482	(0.379–0.613)	1.168	(0.894–1.526)	1.068	(0.796–1.433)	0.987	(0.690–1.410)	1.092	(0.806–1.479)	1.695	(1.198–2.399)
Other	1.609	(1.343–1.927)	0.861	(0.709–1.046)	0.772	(0.643–0.927)	0.786	(0.544–1.137)	0.824	(0.656–1.034)	0.796	(0.571–1.110)
Uninsured	3.656	(3.255–4.106)	0.624	(0.568–0.685)	0.497	(0.443–0.558)	0.647	(0.542–0.772)	0.688	(0.621–0.761)	0.914	(0.771–1.083)
Past-Year Substance Abuse or Dependence												
Alcohol	1.032	(0.946–1.125)	1.163	(1.065–1.271)	0.882	(0.797–0.977)	1.029	(0.896–1.182)	0.975	(0.882–1.077)	0.670	(0.358–1.254)
Pain Relievers	1.005	(0.846–1.195)	1.359	(1.142–1.619)	0.708	(0.571–0.878)	0.710	(0.523–0.964)	0.853	(0.716–1.015)	0.601	(0.226–1.595)
Cocaine	0.926	(0.742–1.156)	1.186	(0.955–1.474)	1.077	(0.804–1.444)	0.628	(0.408–0.967)	0.821	(0.612–1.101)	1.135	(0.904–1.425)
Hallucinogens	1.043	(0.736–1.479)	1.056	(0.754–1.478)	1.072	(0.722–1.592)	0.815	(0.431–1.542)	0.773	(0.547–1.092)	0.694	(0.297–1.624)
Heroin	1.072	(0.763–1.505)	0.515	(0.378–0.703)	0.947	(0.591–1.516)	0.704	(0.400–1.241)	1.029	(0.703–1.506)	0.855	(0.498–1.468)
Inhalants	1.200	(0.674–2.139)	0.617	(0.317–1.198)	0.816	(0.420–1.583)	0.598	(0.278–1.286)	0.636	(0.308–1.316)	1.690	(1.069–2.671)
Marijuana	1.036	(0.921–1.165)	0.960	(0.834–1.105)	0.886	(0.766–1.026)	0.965	(0.801–1.164)	0.800	(0.705–0.909)	0.670	(0.358–1.254)
Sedatives	0.921	(0.525–1.616)	1.537	(0.855–2.765)	0.676	(0.409–1.117)	0.967	(0.475–1.970)	0.550	(0.328–0.924)	0.601	(0.226–1.595)
Stimulants	0.996	(0.762–1.302)	1.647	(1.182–2.296)	0.598	(0.450–0.794)	1.140	(0.726–1.788)	1.122	(0.768–1.639)	1.135	(0.904–1.425)
Tranquilizers	1.063	(0.783–1.443)	1.272	(0.904–1.790)	0.780	(0.571–1.066)	0.980	(0.645–1.489)	0.918	(0.648–1.302)	0.694	(0.297–1.624)

Cost was more likely to be cited as a reason for unmet need by subjects between 26 and 49 years of age, those reporting fair, poor, or very poor self-rated health, and those with who reported being uninsured. Those living in suburban or rural areas were more likely to indicate perceived stigma and minimisation as reasons for unmet need. Respondents with fair, poor, or very poor self-rated health or who reported past year abuse or dependence on either alcohol or pain relievers were also more likely to cite perceived stigma as a reason for unmet need. Respondents with an annual household income of $50,000–$74,999 were more likely to indicate low perceived effectiveness of treatment as a reason for unmet need. In addition, respondents aged 50 and over, females, non-Hispanic Asian respondents, and respondents with at least some high school education were more likely to cite structural barriers as a reason for unmet need.

Several groups had higher odds of reporting reasons other than those shown above as a cause for unmet need: respondents aged 26–49, non-Hispanic mixed respondents, those not working full-time as well as respondents insured by Tricare or VA or those reporting tranquilizer abuse Notably, those with at least some high school education showed much higher odds of reporting a reason not listed above as a cause of unmet need than those with an elementary school education.

## Discussion

Our analyses have elucidated the major characteristics associated with increased odds of unmet need, which include: past year substance abuse or dependence (other than hallucinogens and sedatives), fair, poor, or very poor health, being female, and an educational attainment of college or higher. With respect to reasons for unmet need, cost was most often cited, followed by perceived stigma, structural barriers, and minimisation. Characteristics associated with increased odds of indicating cost as a reason for unmet need include: being uninsured or aged 26–35. Minimisation and low perceived effectiveness are mentioned by high-income persons as reasons for unmet need. College-educated persons and women had higher odds of citing structural barriers as a reason for unmet need.

Our study has some limitations such as using household survey data to assess unmet need as well as demographic, socioeconomic, and health characteristics of the sample population. Perceived unmet need is also a self-reported variable which was not validated using psychiatric diagnostic information; consequently, underreporting or over-reporting of perceived unmet need would affect the accuracy of prevalence estimates for unmet need. Indeed, perceived unmet need is subjective, based on sociocultural factors such as patient expectations and Allin and Masseria suggest that analyses of unmet need are contingent upon the specific phrasing of questions [22]. Small samples for specific subpopulations in this study limit the ability to identify specific patterns of unmet need in these populations. Moreover, the NSDUH excludes individuals with no fixed household address and those living in institutional premises, such as prisons, precluding conclusions on unmet need among these vulnerable populations. In addition, given the repeated, cross-sectional nature of the NSDUH, we are not able to conduct analyses of individuals over time and, therefore, we are unable to determine causality between unmet need and the demographic, socioeconomic, and health correlates under examination. Indeed, there are a number of other potential reasons for and contributors to unmet need which are not examined in this study which bear further scrutiny and study. Nevertheless, the NSDUH has been shown to provide comparable findings to other validated health studies such as the National Comorbidity Survey Replication (NCS-R) and is the only source of data in the United States which provides information on unmet need for a nationally representative sample of adults living in the United States [23]. Our results are consistent with recent and established literature which identify disparities in expressed unmet need based upon age [24], gender [25, 26], economic disadvantage [27], urban/rural status [28], health insurer [29], illicit substance use [30].

## Conclusion

This study extends our understanding of disparities in mental health treatment by not only considering demographic, socioeconomic, and health characteristics of those expressing unmet need from 2002 to 16 but also identifying how these correlate with reasons for unmet need. Cost was a major cause of unmet need among respondents aged 26–49 and those who were uninsured; though the ACA has attempted to reduce the number of uninsured young adults, provisions only guarantee continued enrolment of dependent children until age 25 and, consequently, this may partly explain this observed pattern of expressed unmet need due to cost among these subgroups [31]. Of the reasons for unmet need examined in this study, only perceived stigma and minimisation appear to increase the odds of expressed unmet need among those living in suburban or rural areas, controlling for other demographic, socioeconomic, and health characteristics, highlighting a potential opportunity to develop health promotion interventions for these subpopulations to address unmet need consistent with established literature [32–35]. That the odds of indicating structural barriers as a reason for unmet need were not statistically significantly higher among those living in suburban or rural areas is somewhat surprising, given existing research which indicate that availability of adequate mental health care is a cause for concern [34, 36]. This, in turn, suggests that further research is needed to fully understand the availability or lack thereof of mental health treatment in suburban or rural areas which can inform new initiatives focused on access, such as telemedicine approaches [37, 38]. In addition, the large number of subpopulations expressing unmet need for reasons not specified in this study (i.e. cost, perceived stigma, minimisation, low perceived effectiveness of treatment, and structural barriers) suggests a need to investigate the reasons why these subpopulations express unmet need to address the causal factors underlying why these subpopulations do not receive adequate mental health care

## Data Availability

The datasets supporting the conclusions of this article are available in the Substance Abuse & Mental Health Data Archive repository, https://www.datafiles.samhsa.gov/study-series/national-survey-drug-use-and-health-nsduh-nid13517.
